# Enhancer of Zeste Homolog 2 Protects Mucosal Melanoma from Ferroptosis via the KLF14-SLC7A11 Signaling Pathway

**DOI:** 10.3390/cancers16213660

**Published:** 2024-10-30

**Authors:** Haizhen Du, Lijie Hou, Huan Yu, Fenghao Zhang, Ke Tong, Xiaowen Wu, Ziyi Zhang, Kaiping Liu, Xiangguang Miao, Wenhui Guo, Jun Guo, Yan Kong

**Affiliations:** 1 Key Laboratory of Carcinogenesis and Translational Research (Ministry of Education/Beijing), Department of Renal Cancer and Melanoma, Peking University Cancer Hospital and Institute, Beijing 100142, China; 2111110621@stu.pku.edu.cn (H.D.); 2211210614@stu.pku.edu.cn (L.H.);; 2 Key Laboratory of Carcinogenesis and Translational Research (Ministry of Education/Beijing), Department I of Thoracic Oncology, Peking University Cancer Hospital and Institute, Beijing 100142, China; 3 Department of Life Sciences, Imperial College, London SW7 2AZ, UK

**Keywords:** mucosal melanoma, *EZH2*, ferroptosis, *SLC7A11*

## Abstract

Background: Mucosal melanoma (MM) is epidemiologically, biologically, and molecularly distinct from cutaneous melanoma. Current treatment strategies have failed to significantly improve the prognosis for MM patients. This study aims to identify therapeutic targets and develop combination strategies by investigating the mechanisms underlying the tumorigenesis and progression of MM. Methods: We analyzed the copy number amplification of enhancer of zeste homolog 2 (*EZH2*) in 547 melanoma patients and investigated its correlation with clinical prognosis. Utilizing cell lines, organoids, and patient-derived xenograft models, we assessed the impact of *EZH2* on cell proliferation and sensitivity to ferroptosis. Further, we explored the mechanisms of ferroptosis resistance associated with *EZH2* by conducting RNA sequencing and chromatin immunoprecipitation sequencing. Results: *EZH2* copy number amplification was closely associated with malignant phenotype and poor prognosis in MM patients. *EZH2* was essential for MM cell proliferation in vitro and in vivo. Moreover, genetic perturbation of *EZH2* rendered MM cells sensitized to ferroptosis. Combination treatment of *EZH2* inhibitor with ferroptosis inducer significantly inhibited the growth of MM. Mechanistically, *EZH2* inhibited the expression of Krüpple-Like factor 14 (*KLF14*), which binds to the promoter of solute carrier family 7 member 11 (*SLC7A11*) to repress its transcription. Loss of *EZH2* therefore reduced the expression of *SLC7A11*, leading to reduced intracellular *SLC7A11*-dependent glutathione synthesis to promote ferroptosis. Conclusion: Our findings not only establish *EZH2* as a biomarker for MM prognosis but also highlight the *EZH2-KLF14-SLC7A11* axis as a potential target for MM treatment.

## Simple Summary

Mucosal melanoma is a rare and aggressive form of melanoma that is different from the more common skin melanoma. Unfortunately, current treatments have not significantly improved outcomes for patients with this disease. Our research focuses on understanding the biological mechanisms that drive mucosal melanoma and finding new treatment strategies. We discovered that the enhancer of zeste homolog 2 (EZH2) plays a crucial role in promoting the growth of mucosal melanoma cells and contributes to their resistance to a type of cell death known as ferroptosis. By inhibiting EZH2 and combining this with drugs that induce ferroptosis, we were able to effectively slow tumor growth. Our findings suggest that targeting the EZH2 pathway could offer a new therapeutic approach to treating mucosal melanoma, potentially improving patient outcomes.

## 1. Introduction

Mucosal melanoma (MM), an uncommon melanoma subtype in Western countries, comprises only 1.3% of all melanoma cases [1]. However, in China, it is the second most common subtype, accounting for 20–25% of all melanomas [2,3]. MM predominantly arises in the oral and nasal cavities, as well as the gastrointestinal and genitourinary tracts [4]. Unfortunately, the 5-year survival rate for MM is alarmingly low, at only 25%, which is significantly lower than the 50–80% survival rate for cutaneous melanoma (CM) [2,3,5]. MM has a higher propensity for regional or distant metastasis and recurrence following surgical resection compared to CM [2,6]. Therefore, investigating the molecular mechanisms underlying MM is crucial for improving therapeutic strategies.

Somatic mutations, along with copy number variations, are major contributors to genetic diversity in tumors [7,8]. Recently, several genomic analyses have shown that MM displays a low mutation burden but a higher rate of copy number alterations than CM [9,10]. These molecular differences could cause varying responses to standard treatment between these two melanoma subtypes. The development of targeted therapies and immunotherapies in recent years has dramatically advanced the clinical management of CM, but less so for MM [6,11,12,13]. The majority of patients with metastatic MM have been found to be resistant to these therapies, and their initial response to these therapies generally lasts only for a brief period [14,15,16,17].

The enhancer of the zeste homolog 2 (*EZH2*) gene, located on chromosome 7q36.1, encodes a core component of the polycomb repressive complex 2 (PRC2), which catalyzes histone H3 lysine 27 trimethylation (H3K27me3) [18,19], inducing chromatin compaction and preventing the transcription of target genes [18,20]. In addition to PRC2-based mechanisms, non-PRC2 factors are also recruited or bound by EZH2 to adjust gene expression during oncogenesis [21,22]. Dysregulation of the *EZH2* gene has been observed in various cancer types, including lymphoma, breast, and prostate cancer [23,24,25], which implies that *EZH2* is a promising anticancer target. Emerging evidence has shown that *EZH2* was a key player in promoting CM progression and was associated with poor prognosis [26]. However, the molecular mechanisms underlying the specific function of *EZH2* dysregulation in MM still remain unclear.

Ferroptosis is an iron-dependent cell death type that is often induced by depletion of reduced glutathione (GSH) or massive lipid peroxidation [27]. As a pivotal type of cell death, ferroptosis regulation presents a promising therapeutic strategy for cancer treatment [28]. Ferroptosis can be negatively regulated by solute carrier family 7 member 11 (*SLC7A11*) and glutathione peroxidase 4 (*GPX4*) [29,30,31]. *SLC7A11* imports extracellular cystine into the cell [32], which is subsequently converted to GSH. Recent studies have shown that cancer cells, including melanoma, heavily depend on *SLC7A11* to resist ferroptosis by promoting GSH synthesis, which detoxifies reactive oxygen species (ROS) [33]. In prior studies, *EZH2* inhibited erastin-induced ferroptosis in tongue squamous cell carcinoma (TSCC) through the upregulation of *SLC7A11* [34]. Additionally, *EZH2* has been shown to regulate glutaminase (GLS) expression and promote GSH production in colorectal cancer cells, thereby protecting cells from ferroptosis under metabolic stress [35]. Moreover, polyunsaturated fatty acids in phospholipids are prone to oxidation by redox-active iron, further promoting ferroptosis [36].

In this study, we analyzed the copy numbers of *EZH2* in 547 melanoma tissue samples and discovered that *EZH2* amplification is associated with poor prognosis of MM patients. In MM cell lines and cell-derived xenograft (CDX) models, *EZH2* depletion inhibited MM cell proliferation and tumor growth while sensitizing MM cells to ferroptosis. Mechanistically, *EZH2* represses Krüpple-Like factor 14 (*KLF14*) to activate *SLC7A11* expression. Loss of *EZH2* impedes *SLC7A11*-dependent intracellular GSH synthesis to promote ferroptosis. Overall, our study reveals a novel regulatory axis involved in ferroptosis sensitivity and positions *EZH2* as a promising therapeutic target for MM treatment.

## 2. Materials and Methods

### 2.1. Patients and Tumor Tissue Samples

A total of 547 formalin-fixed, paraffin-embedded (FFPE) tissues were gathered for this retrospective study from Peking University Cancer Hospital (from January 2006 to December 2020). All clinical and pathological data were obtained by medical record review, including age, gender, ulceration, thickness (Breslow), TNM (tumor-node-metastases) stage, and survival (follow-up persisted until October 2022 or until the missing follow-up or death of patients). The approval for this study was granted by the Medical Ethics Committee of the Beijing Cancer Hospital & Institute (approval code: 2019KT92).

### 2.2. QuantiGene Plex DNA Assay

The detailed procedures for immunohistochemistry staining have been previously described [37]. To determine the *EZH2* copy numbers in the samples, the values obtained from the samples were divided by the values from the controls. A copy number of 2 or less was classified as no gain, while a copy number greater than 2 indicated the presence of gain.

### 2.3. Immunohistochemistry

The detailed procedures of immunohistochemistry staining were described previously [38]. Immunohistochemistry analyses were performed using antibodies against EZH2 (5246, Cell Signaling Technology, Danvers, MA, USA) and SLC7A11 (ab37185, Abcam, Cambridge, UK). Three pathologists each independently determined the intensity and density of staining. The scoring method is as follows: The staining intensity is divided into 4 levels as follows: “0” indicates negative; “1”, “2”, and “3” indicate gradual enhancement of positive intensity; and “3” indicates the strongest. The percentage of staining positive cells ranged from 0 to 100%. Histochemistry score (H-score) = percentage of positive cells × staining intensity score. H-score ranges from 0 to 300, and the higher the score, the stronger the staining.

### 2.4. Cell Lines and Cell Culture

HMV-II and GAK cells were obtained from Sigma (St. Louis, MO, USA) and the Japanese Collection of Research Bioresources (JCRB) Cell Bank (Osaka, Japan). LM-MEL-53, HMY1, DEOC1, WM-266-4, SK-MEL-5, A2058, A375, and A875 were purchased from the American Type Culture Collection (ATCC, Manassas, VA, USA). HMV-II cells were cultured in F10 (Gibco, Life Technologies, Grand Island, NY, USA) containing 10% fetal bovine serum (AusGENEX, Brisbane, Australia) and 1% penicillin-streptomycin (Gibco, Life Technologies) at 37 °C with 5% CO_2_. GAK cells were cultured in F12 (Gibco, Life Technologies) containing 10% fetal bovine serum (AusGENEX, Brisbane, Australia) and 1% penicillin-streptomycin (Gibco, Life Technologies) at 37 °C with 5% CO_2_. LM-Mel-53 cells were cultured at 37 °C in 1640 (Gibco, Life Technologies) containing 10% fetal bovine serum (AusGENEX, Brisbane, Australia) and 1% penicillin-streptomycin (Gibco, Life Technologies) at 37 °C with 5% CO_2_. A375, A875, WM 2664, WM 115, A2058, SK-MEL-1, WCF, and MCF-7 cell lines were cultured at 37 °C in DMEM (Gibco, Life Technologies) containing 10% fetal bovine serum (AusGENEX, Brisbane, Australia) and 1% penicillin-streptomycin (Gibco, Life Technologies) at 37 °C with 5% CO_2_.

### 2.5. EZH2 Gain Analysis

Genomic DNA was extracted from the cell lines using a QIAamp DNA kit (Qiagen, Hilden, Germany). Quantitative real-time PCR analysis was conducted on the ABI 7500 FAST real-time PCR system (Applied Biosystems, Foster City, CA, USA), adhering to a previously established protocol [39], with ribonuclease P (RNase P) as the reference gene. Following this, copy numbers were then determined employing the comparative Ct (ΔΔCt) approach of the CopyCaller v2.0 software (Applied Biosystems).

### 2.6. Cell Transient Transfections

pCMV3-HA vector containing the human *EZH2* coding sequence (HA-*EZH2*) was purchased from Sino Biological (HG11337-CY; Sino Biological, Beijing, China). *KLF14* expression plasmids or pCMV6-Myc-DDK vector were purchased from OriGene (RC213087 and PS100001; OriGene, Rockville, MD, USA). *KLF14* siRNA and nontargeting negative control siRNA were obtained from RiboBio (SIGS0015584-1; RiboBio, Guangzhou, China). HMV-II and LM-Mel-53 cells were plated at a density of 2.5 × 10^5^ cells/well in a 6-well plate. After 12–24 h, the cells were transfected using Lipofectamine 3000 reagent (Life Technologies, Carlsbad, CA, USA) according to the manufacturer’s instructions. After 6 h of transfection, the cells were washed and recovered overnight in a fresh medium containing 10% FBS for 48 h. Then, cells were collected for further analysis.

### 2.7. Construction of Lentivirus and Stable Cell Lines

To probe the function of *EZH2*, cells were transfected with lentiviral particles encoding shRNA specifically targeting *EZH2* (shRNA-*EZH2*) and scrambled control shRNA as negative control (shRNA) (Genecoopia, Guangzhou, China). Cells were infected at approximately 70% confluence. After 72 h, 1 µg/mL puromycin (ST551; Beyotime, Shanghai, China) was added to construct stable cell lines, which were then cultured for further assays. The efficacy of *EZH2* knockdown was validated by Western blot and real-time quantitative reverse transcription polymerase chain reaction (qRT-PCR) assays.
h*EZH2* shRNA-1 target sequenceCAGGATGGTACTTTCATTGAAh*EZH2* shRNA-2 target sequenceGTGCAGCTTTCTGTTCAACTTnegative control sequenceTTCTCCGAACGTGTCACGT

### 2.8. RNA Extraction and qRT-PCR

Total RNA was extracted from the cells using TRIzol reagent (Life Technologies) according to the manufacturer’s instructions. Using the ReverTra Ace^®^ qPCR RT Master Mix (FSQ-101, TOYOBO, Osaka, Japan), total RNAs were reverse transcribed. Quantitative real-time polymerase chain reaction (qRT-PCR) was conducted using an ABI 7500 FAST real-time PCR system (Applied Biosystems) and SYBR Green Real-time PCR Master Mix (QPK-201, TOYOBO, Osaka, Japan). *GAPDH* was used as a loading control, and assays were carried out three times.
*EZH2* forward primersGACGGCTTCCCAATAACAGTAG*EZH2* reverse primersTTTGACACCGAGAATTTGCTTC*SLC7A11* forward primersTGTGTGGGGTCCTGTCACTA*SLC7A11* reverse primersCAGTAGCTGCAGGGCGTATT*GAPDH* forward primersCCAGAACATCATCCCTGCCTCT*GAPDH* reverse primersCCTGCTTCACCACCTTCTTGAT

### 2.9. Western Blot

Total cellular and tissue proteins were extracted by adding RIPA buffer (Solarbio, Beijing, China) containing protease inhibitors (Solarbio). The protein concentration was determined using a BCA protein assay kit (Thermo Fisher Scientific, Waltham, MA, USA). Proteins were subsequently separated with 10% SDS-PAGE and transferred to PVDF membranes (Millipore, Burlington, MA, USA). The membranes were blocked with 5% nonfat milk in TBST for 1 hour and then incubated overnight at 4 °C with primary antibodies against EZH2 (5246, Cell Signaling Technology), GAPDH (5174, Cell Signaling Technology), SLC7A11 (A2413, ABclonal, Wuhan, China), KLF14 (A18607, ABclonal). The next day, the membranes were incubated with HRP-linked secondary antibody (7074, Cell Signaling Technology) for 1 h at room temperature. Finally, ECL reagents were used to detect the protein expression (Millipore).

### 2.10. Cell Viability Assay

For the cell growth assay, HMV-II, GAK, and LM-Mel-53 stable cells were seeded into 96-well plates. Cells were cultured for 5 days and were counted every 24 h.

For viability assay, cells were seeded in a 96-well plate. After 12 h, staurosporine (HY-15141, MedChemExpress, Monmouth Junction, NJ, USA), actinomycin D (HY-17559, MedChemExpress), erastin (S7242, Selleck Chemicals, Houston, TX, USA), and ferrostatin-1 (S7243, Selleck Chemicals) were added into medium. Cells were cultured for 48 h. Cell viability was assessed with a CCK-8 assay (CK04, DOJINDO, Beijing, China). A total of 10 μL of CCK8 was added to per well and incubated for 2 h. Then, the absorbance value was measured at 450 nm by a multifunctional plate reader (Infinite 200 Pro, Tecan, Männedorf, Switzerland).

### 2.11. Immunofluorescence

Cells were seeded on glass coverslips in 12-well plates at a density of 1.5 × 10^5^ cells/well and allowed to adhere for 24 h. For staining, cells were washed with PBS and fixed with 4% paraformaldehyde for 15 min at room temperature. After fixation, cells were permeabilized with 0.1% Triton X-100 in PBS for 10 min and blocked with 3% BSA in PBS for 1 h. Primary antibodies against Ki-67 (9449, Cell Signaling Technology, 1:2000) were applied overnight at 4 °C. Then, cells were washed three times with PBS and incubated with fluorescently labeled secondary antibodies (715-585-150, Jackson ImmunoResearch, West Grove, PA, USA, 1:500) for 1 h at room temperature in the dark. Nuclei were labeled with DAPI for 5 min. Finally, fluorescence images were captured using a Leica DMi8 confocal microscope.

### 2.12. RNA-Seq

Total RNA from HMV-II cells with *EZH2* knockdown was evaluated using the Bioanalyzer 2100 system (Agilent Technologies, Santa Clara, CA, USA). mRNA was purified and library fragments were processed with the AMPure XP system (Beckman Coulter, Brea, CA, USA), followed by PCR amplification. The library insert size was assessed, and sequencing was performed on the Illumina NovaSeq 6000. Reads were counted and FPKM values were calculated using FeatureCounts (v1.5.0-p3) and DESeq2 R package (1.20.0). Significant differential expression was determined with thresholds of *p* < 0.05 and |log2(foldchange)| > 0.585.

### 2.13. Reactive Oxygen Species (ROS) Detection

Cells were seeded in 6-well plates and loaded with 5 μm CellROX Deep Red Reagent fluorogenic probe (Molecular Probes, Life Technologies) by incubating them at 37 °C for 30 min. The medium was removed and cells were washed three times with PBS, digested with trypsin to obtain cell pellets, and suspended with 200 μL PBS. Samples were analyzed using a flow cytometer CytoFLEX (Beckman Coulter, Brea, CA, USA) at excitation/emission of 638/660 nm. ROS production was estimated using the mean fluorescence intensity of the cell population by FlowJo software (v 10.8.1).

### 2.14. MDA Measurements

We used the Micro MDA Assay Kit (BC0025, Solarbio, Beijing, China) to evaluate the level of lipid oxidation according to the manufacturer’s instructions. We calculate the MDA concentration with a multifunctional plate reader (Infinite 200 Pro, Tecan).

### 2.15. GSH Assay

GSH levels were measured using a GSH-Glo Glutathione Assay kit (V6911, Promega, Madison, WI, USA). In brief, cells were seeded into 96-well plates at 8000 cells per well. After overnight incubation, we carefully removed the culture medium from the wells, and 100 μL of prepared 1× GSH-GLO Reagent was added to each well, mixed briefly on a plate shaker, and incubated for 30 min at room temperature. Then, 100 μL of reconstituted Luciferin Detection Reagent was added to each well, mixed briefly on a plate shaker, and incubated for 15 min. Luminescence was detected by a multifunctional plate reader (Infinite 200 Pro, Tecan).

### 2.16. TEM

Stably *EZH2*-depleted or control LM-Mel-53 cells were fixed with 2.5% glutaraldehyde (P1126, Solarbio, Beijing, China) for 30 min, and post-fixed in 1% OsO4 for 2 h at 4 °C. After washing 3 times with PBS, the samples were sequentially dehydrated, permeabilized, embedded, polymerized, and cut into sections. Sections were stained with uranium acetate and lead citrate and then dried overnight. The TEM image was obtained under an electron microscope (HT7700, Hitachi, Tokyo, Japan).

### 2.17. ChIP-Seq

ChIP assays were performed using the SimpleChIP Enzymatic Chromatin IP Kit (9003, Cell Signaling Technology) following the manufacturer’s instructions. HMV-II cells with *EZH2* knockdown were fixed with formaldehyde, and chromatin was digested to 150–900 bp fragments with Micrococcal Nuclease. ChIP was conducted using anti-EZH2 antibody (5246, Cell Signaling Technology) and ChIP-Grade Protein G Magnetic Beads (9006, Cell Signaling Technology). DNA was purified and then verified on an agarose gel, and the library was constructed by Novogene Corporation (Beijing, China). Sequencing was performed on the Illumina platform (Illumina, San Diego, CA, USA), and quality was assessed with the Agilent Bioanalyzer 2100 system. The raw reads from ChIP-Seq were mapped to the human genome (hg19) using BWA mem (v 0.7.12). After aligning the reads to the reference genome, peak calling was performed using MACS2 (v 2.1.1) with a q-value threshold of 0.05 to identify significant peaks [40]. These peaks were then annotated using ChIPseeker, which mapped them to different genomic regions, including promoter, 5′ UTR, 3′ UTR, exons, introns, downstream, and intergenic regions. Motifs were identified with Homer. Next, differential peak analysis was carried out using edgeR to identify ChIP-Seq peaks significantly affected by *EZH2* knockdown. The criteria for significance were set as padj < 0.05 and |log2FoldChange| > 1. Validation was performed with ChIP-qPCR.
*SLC7A11*-intron ForwardGAACCTGACCCTGGGAGAAAAC*SLC7A11*-intron ReverseCTGAAGCTGTGATTTAAGGACTGG*SLC7A11*-promoter ForwardGCAAACCTGGAGAATTTGCATCA*SLC7A11*-promoter ReverseCTTGTATTTAAGCGCCTGCCT*KLF14*-promoter-1 ForwardAACTTTCTGGGACTCCGC*KLF14*-promoter-1 ReverseCCGGCTAAGTCATGTTTA*KLF14*-promoter-2 ForwardTGCAACTTGACAAACTAATGCT*KLF14*-promoter-2 ReverseAAGGACATATCCTCTCTTTGTTCA

### 2.18. Dual-Luciferase Reporter Assay

Cells were either pre-transfected with *KLF14* overexpression or not, and were cotransfected with pGL3-*SLC7A11* or pGL3-*SLC7A11-ΔKLF1* expression vectors using Lipofectamine 3000. Renilla luciferase expression vector (CV045) was also transfected into cells as a transfection control. After 48 hours of transfection, luciferase assays were performed using a Dual-Luciferase Reporter Assay System (E1910, Promega, Madison, WI, USA) following the manufacturer’s instructions. Relative firefly luciferase activity was normalized to the corresponding values of renilla luciferase.
*KLF14* binding siteAAGTTGGTGTGACA

### 2.19. CDX Model and Treatment

The stable *EZH2*-depleted and control HMV-II, GAK cells (5 × 10^6^) were implanted subcutaneously into 5-week-old female NOD-SCID mice. Tumor size and mouse weight were measured every 3 days and tumor volume was calculated using the following formula: volume = length × width^2^/2. At the end of 15 days, mice were sacrificed by cervical dislocation. All animal care and experimental procedures were carried out in accordance with the Animal Care Ethics guidelines approved by the Medical Ethics Committee of Beijing Cancer Hospital & Institute.

### 2.20. PDX Model and Treatment

PDX models were established as described previously [37]. The mice were treated daily with MS8815 (20 mg/kg, i.p., daily), erastin (20 mg/kg, i.p., daily), or vehicle for 18 days. Tumor size and mouse body weight were measured every 3 days, and tumor volume was calculated using the following formula: volume = length × width^2^/2. The mice were sacrificed by cervical dislocation and analyzed after 18 days of administration. Tumors were preserved for immunohistochemistry examination at the endpoint in 10% formalin. All animal procedures followed the Animal Care Ethics guidelines with the approval of the Medical Ethics Committee of Beijing Cancer Hospital & Institute.

### 2.21. Statistical Analyses

Continuous data, such as age, were expressed as means ± standard deviations (SD) for normally distributed variables. Correlations between aberration status and clinical parameters were assessed using the Chi-square test or Fisher’s exact test. OS curves were generated using the Kaplan–Meier method and compared between groups with the log-rank test. Cox proportional hazard models were used to calculate hazard ratios (HRs) and 95% confidence intervals (CIs). All statistical analyses were conducted using SPSS version 24 (IBM, Armonk, NY, USA) and GraphPad Prism version 9 (GraphPad Software, San Diego, CA, USA). All tests were two-sided, and a *p* value of less than 0.05 was considered statistically significant.

## 3. Results

### 3.1. Copy Number Amplification of EZH2 Correlates with Poor MM Prognosis

**Figure 1:**
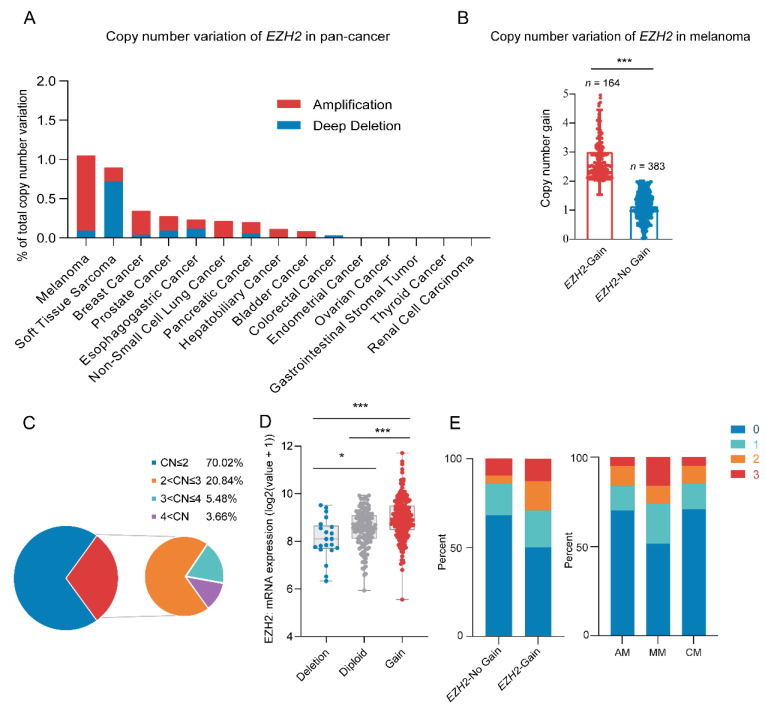
Copy number variation of *EZH2* gene in melanoma. (**A**) *EZH2* gain status in different cancer types from the cBioPortal database. (**B**) Copy number variation of *EZH2* gene in 547 melanoma samples. *** *p* < 0.001. (**C**) Distribution pie chart of *EZH2* copy number (*n* = 547). According to the *EZH2* copy number, it is divided into four subgroups. Subgroup 1: copy number ≤ 2; subgroup 2: 2 < copy number ≤ 3; subgroup 3: 3 < copy number ≤ 4; subgroup 4: copy number > 4. (**D**) Correlation of *EZH2* gain status with its mRNA expression in melanoma samples from the cBioPortal database (*n* = 367). * *p* < 0.05, *** *p* < 0.001. (**E**) Association of *EZH2* copy number gain with expression levels in melanoma subtypes (*n* = 183). The left panel represents the percentage of cases with or without *EZH2* copy number gain across different expression levels (0, 1, 2, 3). The right panel illustrates the proportion of expression levels in different melanoma subtypes: acral melanoma (AM), mucosal melanoma (MM), and cutaneous melanoma (CM). The staining score for each sample, counting the intensity of the staining, was graded as 0, 1, 2, and 3 (“0” as negative, and “3” as the strongest).

Through analyzing the pan-cancer copy number variations of the *EZH2* gene using the cBioPortal database [41,42], we identified a high frequency of *EZH2* gene copy number amplification in melanoma [43] (Figure 1A). Next, we examined the copy number of the *EZH2* gene in 547 melanoma samples. Among a total of 164 (30.0%) samples that showed amplification of the *EZH2* gene, 2–3 copies were the most common copy number distribution, accounting for 20.84% (Figure 1B,C). Notably, the copy number amplification rate of the *EZH2* gene in MM was significantly higher than that in other melanoma subtypes (*p* < 0.001) (Table 1).

Among 547 melanoma patients, MM showed higher *EZH2* gain frequency, significant only in the primary site (64/164,39.0%, *p* < 0.001). Detailed information and statistical results are shown in Supplementary Table S3. Furthermore, correlations between *EZH2* gain and clinicopathological features were specifically performed in the MM subtype (Table 2). Most of the patients with *EZH2* gain were in stages III and IV (*p* = 0.017). In contrast, other factors such as age, gender, tumor thickness, ulceration, pathogenic site, and coexistence of other therapeutic targets (NRAS, BRAF, and KIT) showed no significant differences in *EZH2* copy number gain. Taken together, *EZH2* gain may predict a more progressive phenotype of MM.

**Figure 2:**
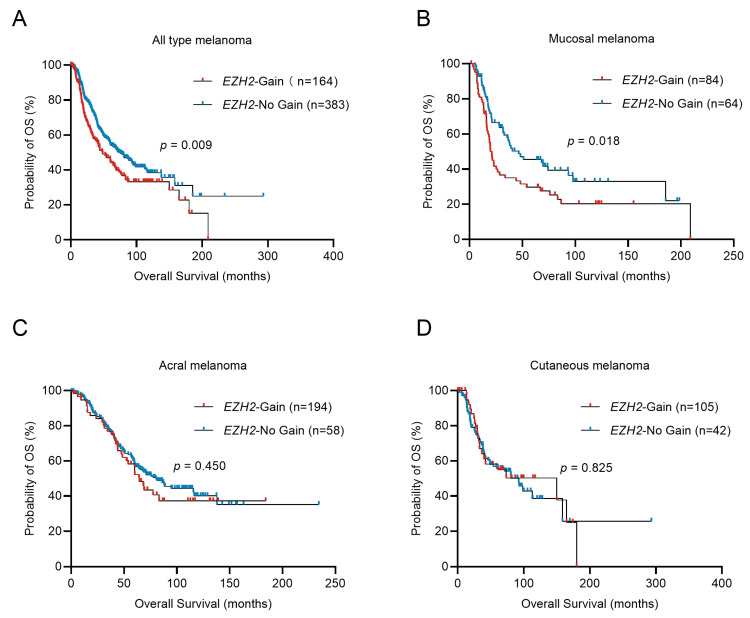
Overall survival of melanoma patients in relation to *EZH2* copy number variations. Comparison of the overall survival (OS) of tumors with different *EZH2* copy number levels in melanoma subtypes was conducted by the Kaplan–Meier method. (**A**) all melanoma cases, *n* = 547. (**B**) acral melanoma cases, *n* = 252. (**C**) cutaneous melanoma cases, *n* = 147. (**D**) mucosal melanoma cases, *n* = 148. *EZH2*-No gain was considered as samples with copy numbers less than or equal to 2.0. *EZH2* gain was considered as samples with copy numbers greater than 2.0.

Survival analysis showed that, in the total cohort, *EZH2* amplification shortened OS in melanoma patients (48.30 vs. 74.30 months; *p* = 0.009, Figure 2A). Stratified analysis indicated only in MM that the median overall survival (OS) time for patients with *EZH2* gain was significantly shorter than that without *EZH2* gain (21.20 vs. 46.63 months; *p* = 0.018, Figure 2B) (in acral melanoma (AM): 64.70 vs. 80.50 months; *p* = 0.450, Figure 2C) (in CM: 149.90 vs. 92.37 months; *p* = 0.825, Figure 2D). In the univariate and multivariate Cox analysis, *EZH2* gain is a poor prognostic factor for MM, independently affecting OS (Table 3). This result indicated that *EZH2* gain may play an essential role in tumorigenesis and progression of MM.

### 3.2. EZH2 Is Required for MM Proliferation In Vitro and In Vivo

Utilizing the Cancer Cell Line Encyclopedia (CCLE) database [44,45], we found a positive correlation between *EZH2* copy number and its expression levels in cells (Supplementary Figure S1A,B). Further analysis in 11 melanoma cell lines revealed that the protein expression levels of EZH2 in cell lines with *EZH2* copy number gain (GAK, LM-MEL-53, and A375) were significantly higher than those within the normal range (SK-MEL-5), though not uniformly across all cell lines (Supplementary Figure S1C,D).

**Figure 3:**
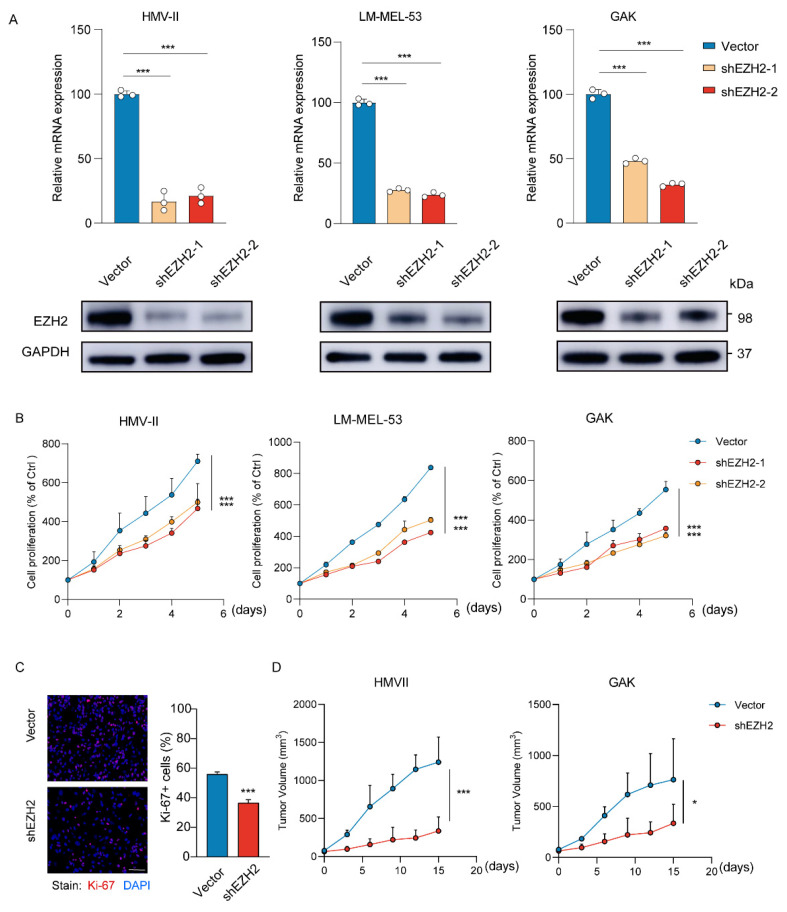
Loss of *EZH2* inhibits MM cell proliferation and progression in vitro and in vivo. (**A**) The expression of EZH2 was detected by RT-qPCR (top row) or Western blot assay (bottom row) after the knockdown of *EZH2*. The data are presented as the mean ± SEM. *n* = 3, *** *p* < 0.001. (**B**) HMV-II, LM-MEL-53, and GAK cells with stable depletion of *EZH2* or control were grown for 5 days, with cell numbers counted every day by CCK-8 assays. The changes in cell numbers were compared to day 0, and the mean ± SEM from 3 experiments was plotted. *** *p* < 0.001. (**C**) The proliferative abilities of stably *EZH2*-depleted HMVII cells were measured with Ki-67 staining assay. Three experiments were conducted with mean ± SEM of percentage of Ki-67-positive cells plotted. Scale bar: 100 μm. *** *p* < 0.001. (**D**) The average sizes of xenograft tumors were measured every 3 days and plotted (*n* = 5, error bars indicate mean ± SEM). * *p* < 0.05, *** *p* < 0.001.

To determine the effects of *EZH2* on MM cells, HMV-II, LM-MEL-53, and GAK cell lines were genetically modified to stably knock down *EZH2* (Figure 3A). Depletion of *EZH2* significantly inhibited the growth of cells (Figure 3B), accompanied by a notable decrease in proliferation (Figure 3C). To further verify the function of *EZH2* in cell growth in vivo, the stable *EZH2*-depleted HMV-II and GAK cell-derived xenograft mouse models were established. Consistent with our observation in vitro, *EZH2*-depleted MM cells grew slower and formed smaller tumors (Figure 3D). These results indicated that *EZH2* actually contributes to MM cell proliferation and may be a potential therapeutic target in MM.

### 3.3. EZH2 Depletion Sensitizes MM Cells to Ferroptosis by Downregulation of SLC7A11

**Figure 4:**
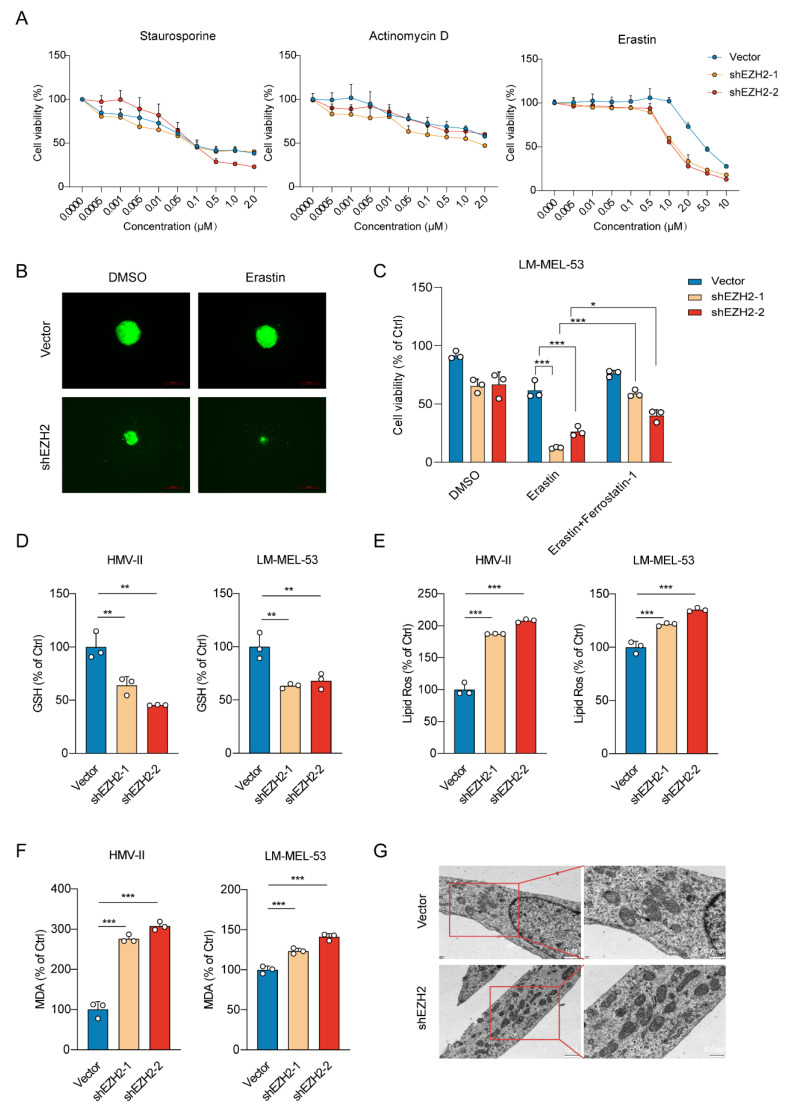
*EZH2* knockdown enhances ferroptotic sensitivity in MM cells. (**A**) Cell viability response to treatment with apoptosis and ferroptosis inducers in cells with *EZH2* knockdown. Cell viability was assessed after treatment with a range of concentrations of apoptosis inducer staurosporine (left panel), apoptosis inducer actinomycin D (middle panel), and ferroptosis inducer erastin (right panel). The mean ± SEM from 3 experiments was plotted. (**B**) Representative images of *EZH2* knockdown effects on the viability of 3D spheroids formed by LM-MEL-53 cells in response to 4 µM erastin, as indicated by GFP fluorescence. Scale bars: 200 µm. (**C**) Bar graph showing viability of LM-MEL-53 cells with *EZH2* knockdown, treated with 4 µM erastin or 4 µM erastin and 4 µM Ferrostatin-1. The data are presented as the mean ± SEM. *n* = 3, * *p* < 0.05, *** *p* < 0.001. (**D**) Bar graph demonstrating intracellular glutathione levels in *EZH2*-depleted HMV-II and LM-MEL-53 cells. The data are presented as the mean ± SEM. *n* = 3, ** *p* < 0.01. (**E**) Lipid peroxidation was measured by flow cytometry after 5 μM CellROX Deep Red staining in *EZH2*-depleted cells. The data are presented as the mean ± SEM. *n* = 3, *** *p* < 0.001. (**F**) The level of malondialdehyde in cells was determined by using a malondialdehyde kit after the knockdown of *EZH2*. The data are presented as the mean ± SEM. *n* = 3, *** *p* < 0.001. (**G**) TEM was used to detect the mitochondrial morphology of ferroptotic cells. Scale bars: 1 μm (left column), and 500 nm (right column).

To investigate the effect of *EZH2* knockdown on cell death mechanisms, we exposed *EZH2*-depleted melanoma cells to various cell death inducers. Our data revealed a substantial elevation in the susceptibility of cells to ferroptosis upon treatment with Erastin, but not to apoptosis or autophagy inducers (staurosporine or actinomycin D), indicating a specific modulation of ferroptosis pathways by *EZH2* (Figure 4A). Similarly, 3D spheroids fluorescence imaging revealed that *EZH2* knockdown enhanced ferroptotic sensitivity upon erastin treatment (Figure 4B). A reduction in *EZH2* significantly promoted erastin-induced cell death, preventable by ferrostatin-1 (Figure 4C). To further investigate the potential role of *EZH2* in ferroptosis, we subsequently examined the levels of GSH. GSH, a vital antioxidant, maintains redox balance and inhibits ferroptosis. *EZH2* knockdown reduced GSH, supporting its role in suppressing ferroptosis (Figure 4D). The knockdown of *EZH2* also notably elevated lipid peroxidation and the generation of Malondialdehyde (MDA) (Figure 4E,F). Transmission electron microscopy (TEM) analysis revealed mitochondrial shrinkage with elevated membrane density, a typical morphologic feature of ferroptosis, in *EZH2*-depleted melanoma cells (Figure 4G). Therefore, loss of *EZH2* enhances the susceptibility of MM cells to ferroptosis.

**Figure 5:**
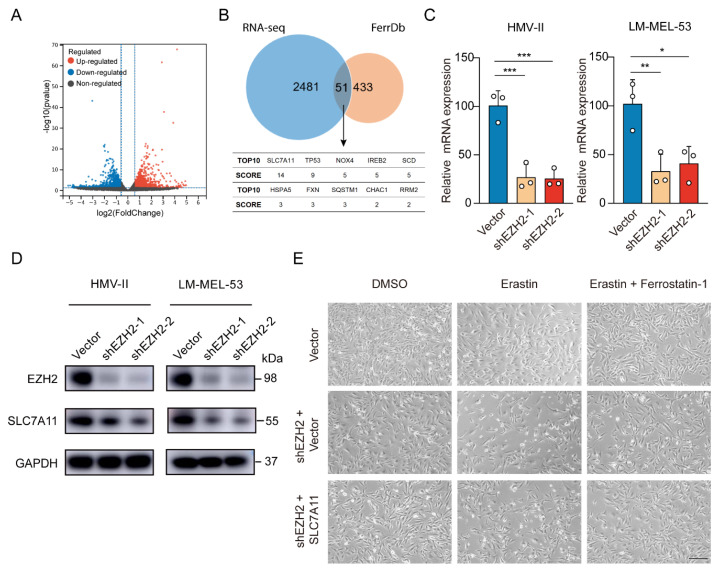
Depletion of *EZH2* stimulates ferroptosis through decreased *SLC7A11*. (**A**) Volcano plot displaying differential expression from RNA-seq data. Red points indicate upregulated genes, blue points show downregulated genes, and grey points represent genes without significant changes. Vertical dashed lines mark fold change thresholds, and the horizontal line indicates the *p*-value cutoff for significance. (**B**) Venn diagram showing the significant overlap between RNA-seq data and FerrDb database. SCORE values were derived from FerrD. The SCORE values were listed below the Venn diagram, with *SLC7A11* as the top hit (**C**,**D**). The expression of *SLC7A11* was detected by RT-qPCR (**C**) or Western blot assay (**D**) with *EZH2* knockdown. The data are presented as the mean ± SEM. *n* = 3, * *p* < 0.05, ** *p* < 0.01, *** *p* < 0.001. (**E**) Representative phase-contrast images of *EZH2*-depleted LM-MEL-53 cells, with or without *SLC7A11* re-expression, treated with 4 μM erastin or 4 µM erastin and 4 µM Ferrostatin-1 (*n* = 3). Scale bar: 200 μm.

To elucidate *EZH2*’s role in mucosal melanoma progression, we also performed RNA sequencing (RNA-seq) analysis, revealing 1182 genes were upregulated and 1350 genes downregulated after *EZH2* depletion (Figure 5A). Comparing *EZH2*-regulated differentially expressed genes (DEGs) from RNA-Seq with the ferroptosis-related genes of FerrDb, we identified 51 overlapping genes, indicating *EZH2*’s pivotal role in ferroptosis. According to the FerrDb database, among the top 10 DEGs, *SLC7A11* had the highest score (related score = 14) (Figure 5B), which is highly related to ferroptosis. With *EZH2* knockdown, *SLC7A11* mRNA and protein levels, a critical suppressor of ferroptosis, dropped significantly (Figure 5C,D). Restoring *SLC7A11* in *EZH2*-knockdown cells nearly abolished erastin-triggered cell death (Figure 5E). Thus, these results suggest *EZH2* inhibits MM progression at least partly through *SLC7A11* suppression and ferroptosis promotion.

### 3.4. EZH2 Promotes SLC7A11 Expression by Repressing KLF14

**Figure 6:**
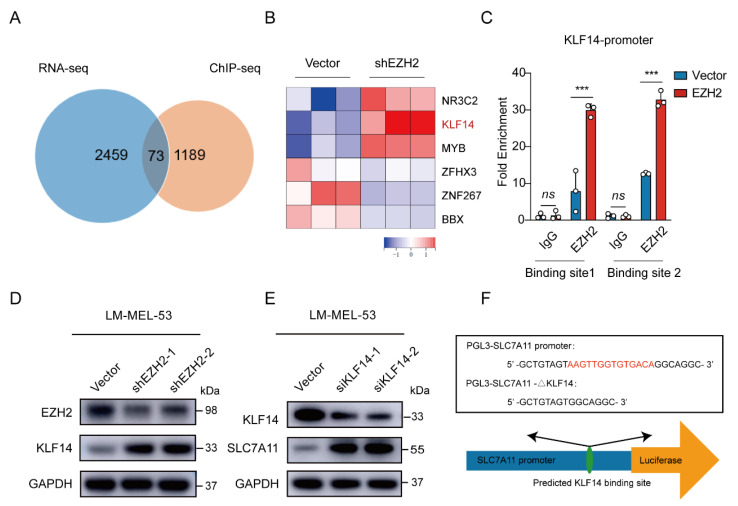

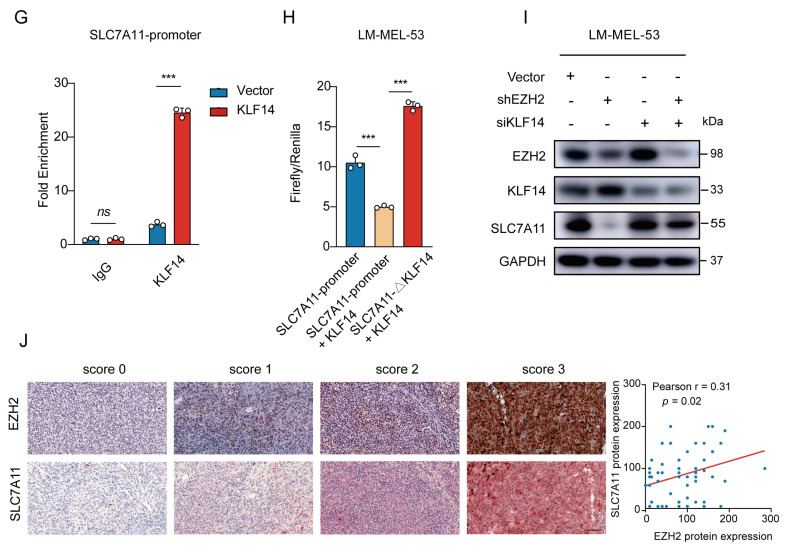
*EZH2*-mediated *SLC7A11* upregulation is regulated by *KLF14*. (**A**) Venn diagram of RNA-seq and ChIP-seq related genes showing that 73 genes were found to be potential target genes of *EZH2*. (**B**) Heatmap showing the 6 transcription factors of the 73 potential target genes of *EZH2*. (**C**) The binding of *EZH2* to *KLF14* promoter was detected after *EZH2* overexpression by ChIP-qPCR. IgG as a negative control. The data are presented as the mean ± SEM. *n* = 3, ns, not significant, *** *p* < 0.001. (**D**) The protein level of KLF14 was detected with *EZH2* depletion. (**E**) The protein level of SLC7A11 after transfection of *KLF14* siRNA. (**F**) Schematic representation of the predicted *KLF14* binding site within the *SLC7A11* promoter. (**G**) The binding of *KLF14* to *SLC7A11* promoter was detected after *KLF14* overexpression by ChIP-qPCR. IgG as a negative control. The data are presented as the mean ± SEM. *n* = 3, ns, not significant, *** *p* < 0.001. (**H**) Luciferase assay measuring *SLC7A11* promoter activity before and after *KLF14* binding site deletion in the absence or presence of *KLF14*. Luciferase activities were normalized to Renilla luciferase activity. The data are presented as the mean ± SEM. *n* = 3, *** *p* < 0.001. (**I**) Western blot analysis of EZH2, KLF14, and SLC7A11 protein expression following dual knockdown of *EZH2* and *KLF14*. (**J**) The correlations between EZH2 and SLC7A11 protein expression in MM patients were analyzed by Pearson correlation analysis (*n* = 55). Representative images from immunohistochemical staining of EZH2 and SLC7A11 protein expression. The staining score for each sample, counting the intensity of the staining, was graded as 0, 1, 2, and 3 (“0” as negative, and “3” as the strongest). Scale bar: 100 μm.

Given that *EZH2* acts as a transcription factor, we performed chromatin immunoprecipitation using EZH2 antibody followed by Next Generation Sequencing (ChIP-Seq) to analyze its regulation of *SLC7A11*. The results showed an *EZH2* binding site in the intron 3 region of *SLC7A11*. ChIP-PCR analysis confirmed an enriched *EZH2* binding site at a putative site in *EZH2*-overexpressed cells (Supplementary Figure S2A). However, when we directly tested whether *EZH2* regulates *SLC7A11* transcription through this binding site, we constructed a transcription reporter with the identified sequence inserted upstream of the *SLC7A11* promoter. Surprisingly, the dual luciferase reporter assay showed that this sequence did not significantly influence *SLC7A11* promoter activity (Supplementary Figure S2B,C), suggesting that *EZH2* may regulate *SLC7A11* indirectly, possibly through transcriptional regulators. To explore this further, we integrated our RNA-Seq and ChIP-Seq datasets. Of the 73 intersecting genes, *KLF14* emerged as one of six transcription factors significantly altered by *EZH2* knockdown (Figure 6A,B). *KLF14* is known to regulate various genes through its DNA-binding zinc finger domains [46] and has been implicated in metabolic processes, including reducing lipid accumulation and oxidative stress [47,48,49]. Previous studies also revealed that *EZH2* binds to the *KLF14* promoter region [50]. To investigate this interaction, we performed ChIP-qPCR, confirming higher *EZH2* enrichment at the *KLF14* promoter in *EZH2*-overexpressing cells (Figure 6C). Meanwhile, the protein level of KLF14 was elevated upon EZH2 knockdown (Figure 6D). Collectively, these results suggest that *EZH2* indirectly regulates *SLC7A11* by repressing *KLF14* transcription through direct binding to the *KLF14* promoter.

To ascertain the regulatory role of *KLF14* on *SLC7A11* expression, a Western blot revealed increased SLC7A11 levels with KLF14 knockdown (Figure 6E). Then, analyzing the *SLC7A11* promoter using the JASPAR database [51] displayed potential binding sites for *KLF14* (Figure 6F). Subsequent ChIP-qPCR assays showed a significant increase in the enrichment of *KLF14* at the promoter region of *SLC7A11* in *KLF14*-overexpressed cells (Figure 6G). Furthermore, luciferase reporter assays showed *KLF14* overexpression inhibited the activity of luciferase reporter *SLC7A11*, reversed by deleting the *KLF14* binding site in the *SLC7A11* promoter (Figure 6H). Moreover, *EZH2* knockdown reduced SLC7A11 expression; however, *KLF14* inhibition attenuated this effect (Figure 6I), indicating that *EZH2* regulates *SLC7A11* through *KLF14.*

To clinically validate this signaling axis, we examined EZH2 and SLC7A11 expression in 55 MM patients by immunohistochemistry staining. Statistically, EZH2 expression was positively correlated with SLC7A11 expression (r = 0.31, *p* = 0.02) (Figure 6J). Together, these results align with previous data, suggesting that elevated EZH2 may contribute to ferroptosis resistance by increasing SLC7A11 levels in MM, pointing to a therapeutic strategy combining EZH2 and ferroptosis targets.

### 3.5. Combination EZH2 Inhibitor and Ferroptosis Inducer Treatment Suppresses Tumorigenesis of MM

**Figure 7:**
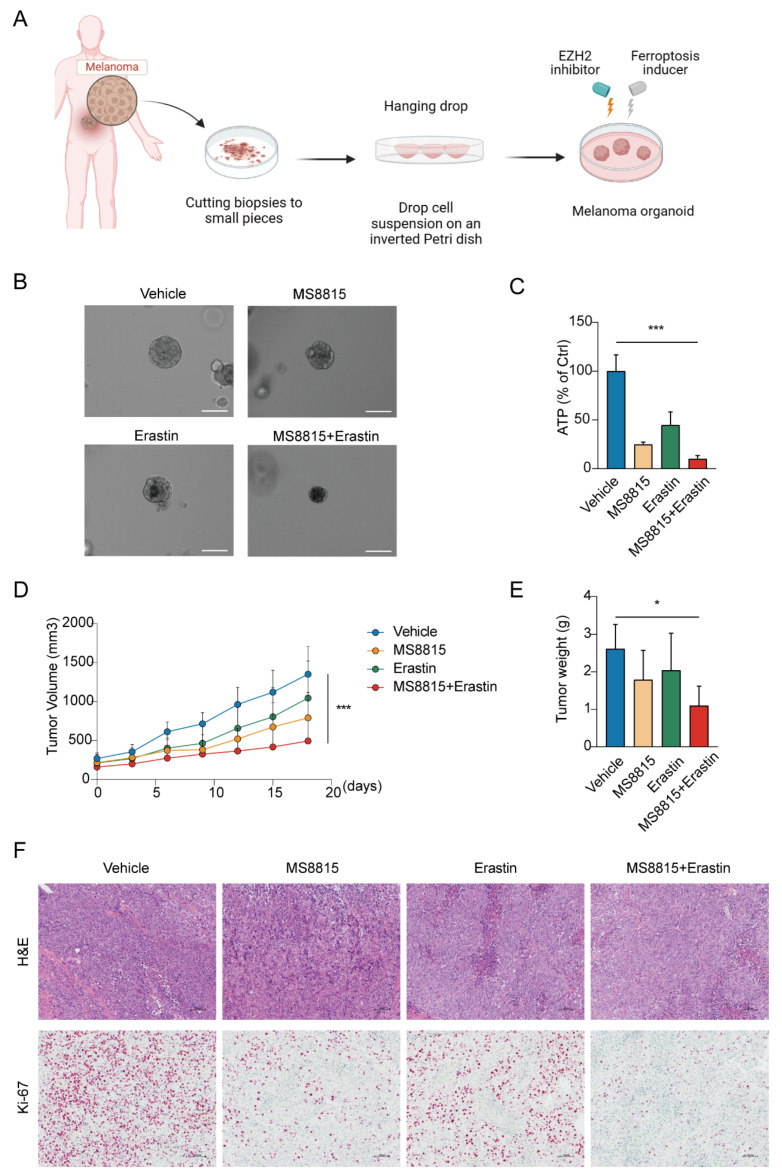
Effects of combined EZH2 inhibitor and ferroptosis inducer treatment in MM. (**A**–**C**) Evaluation of combinatorial treatment effects on melanoma organoid models. (**A**) Schematic representation of the experimental design for combinatorial treatment with MS8815 and erastin on melanoma organoid models. (**B**) Representative images of melanoma organoids treated with DMSO (vehicle control), MS8815, erastin, and a combination of MS8815 and erastin, showing morphological changes (*n* = 3). Scale bar: 100 µm. (**C**) Quantification of cellular ATP levels as a measure of cell viability post-treatment (*** *p* < 0.001 compared to vehicle control). (**D**–**F**) Effects of combined MS8815 and erastin on tumor growth in a MM PDX model. (**D**) Tumor volume was measured over an 18-day period. (**E**) Tumors were weighed and plotted (*n* = 5). Data were expressed as mean ± SEM. * *p* < 0.05, *** *p* < 0.001. (**F**) Representative images from H&E and immunohistochemical staining of Ki-67 protein expression. Scale bars: 100 μm.

We evaluated the therapeutic synergy of EZH2 inhibitor MS8815 and ferroptosis inducer erastin on organoids derived from MM patients (Figure 7A). MS8815 or erastin alone moderately reduced organoid size, but their combination resulted in a significant volume reduction (Figure 7B). Quantitatively, ATP assay confirmed this, with the combined treatment significantly lowering ATP levels (*p* < 0.001, Figure 7C).

To further explore the therapeutic effects of combining an EZH2 inhibitor and a ferroptosis inducer, we employed a patient-derived xenograft model in mice, established with tumors from MM patients. After 18 days of treatment with vehicle, MS8815, erastin, or a combination (Figure 7D–F), we observed significant tumor growth inhibition with the combined therapy, surpassing monotherapy and the control groups (*p* < 0.05).

## 4. Discussion

MM differs epidemiologically and molecularly from CM, challenging the establishment of treatment guidelines due to unclear pathogenesis and lack of intervention targets. Genomics studies reveal MM displays a low mutation burden but a higher rate of copy number alterations than CM [9,10]. During our genome sequencing to search for melanoma driver genes, we previously noted *EZH2* copy number amplification. A study of nearly 700 archival patient tissue samples, encompassing different cancer types including CM and cancers of the endometrium, prostate, and breast, revealed a significant association between *EZH2* expression and high proliferation rate [52]. Previous studies have shown that *EZH2* is crucial in melanoma progression and metastasis [26,53], yet its function in MM remains elusive. Our study investigated *EZH2* copy number variation in 547 melanoma patients, revealing *EZH2* copy number gain in MM specifically. The *EZH2* high-melanoma patient group showed a significantly shorter survival [26]. Consistent with this, the stratified analysis indicated that patients with *EZH2* gain had significantly shorter survival only in MM and not CM or AM. In vitro and in vivo studies also confirmed that high *EZH2* expression contributed to MM proliferation.

Ferroptosis, a cellular death process driven by iron-dependent phospholipid peroxidation [54], is regulated by different pathways found in various cancers [55]. The role of *EZH2* in ferroptosis is currently controversial. It inhibits ferroptosis in TSCC cells by inhibiting miR-125b-5p and enhancing *SLC7A11* [34], while in acute myeloid leukemia (AML), muscle ARNT-Like protein-1 (Bmal1) prevented RSL3-induced ferroptosis through *EZH2*-mediated *EBF3* methylation to inhibit the expression of *EBF3* and *ALOX15* [56]. Meanwhile, mesenchymal stem cell-derived exosomal miR-367-3p could restrain *EZH2* expression to suppress ferroptosis in multiple sclerosis (MS) [57]. HBV X protein (HBx) facilitates ferroptosis in acute liver failure by *EZH2*-mediated *SLC7A11* suppression [58]. Currently, the role of ferroptosis and its regulatory mechanisms by *EZH2* in MM remain largely unknown. Herein, we demonstrated that *EZH2* depletion enhances ferroptosis, lipid peroxidation, and MDA production, and decreases GSH level, sensitizing MM cells to erastin-induced ferroptosis. Mechanically, we revealed a positive correlation between *EZH2* and *SLC7A11* expression.

*SLC7A11*, a cystine transporter, is frequently overexpressed in human malignancies [59]. Its high expression fosters ferroptosis resistance, thereby promoting tumorigenesis and progression [60,61,62,63]. Notably, its overexpression was correlated with melanoma staging and progression, accelerating cell proliferation in vivo and in vitro [63]. Multiple transcription factors could regulate *SLC7A11* expression, including nuclear factor erythroid 2-related factor 2 (*NRF2*) and activating transcription factor 4 (*ATF4*), which were intracellular key antioxidant defense regulators and modulated *SLC7A11* levels to negatively regulate ferroptosis [64,65]. *SOX* also upregulated *SLC7A11* expression, conferring resistance to ferroptosis in lung cancer [66]. Rather than directly regulating *SLC7A11*, we found that *EZH2* upregulation increased *SLC7A11* expression by downregulating *KLF14*, thereby promoting ferroptosis. Our study is the first to report that *KLF14* could bind to the *SLC7A11* promoter, repressing its expression. *KLF14* was significantly downregulated in hepatocellular carcinoma (HCC), correlating with poor prognosis, and inhibits the proliferation of HCC cells by modulating cellular iron metabolism via the repression of Iron-responsive element-binding protein 2 (*IRP2*) [67]. Overexpression of *KLF14* significantly reduced breast cancer cell proliferation and invasion [68]. In pancreatic epithelial cancer, *KLF14* acts as a transcriptional co-repressor with *mSin3A* and *HDAC2* to silence the TGFbeta receptor II promoter [69]. Our current study found that *KLF14* expression was negatively regulated by *EZH2*, while *KLF14* directly represses *SLC7A11* promoter activity, indicating a protective role in MM.

Recent studies have further established that *EZH2* plays a pivotal role in regulating cancer cell metabolism, particularly glutamine metabolism, by inhibiting GLS activity [35]. Glutamine is essential for synthesizing GSH, a key molecule involved in maintaining redox balance and preventing ferroptosis. In pancreatic cancer, glutamine deprivation has been shown to reduce *GPX4* expression, a central regulator of ferroptosis, thus sensitizing cells to ferroptosis through the *KRAS/MAPK-NRF2-GPX4* pathway [70]. This suggests that *EZH2* may regulate ferroptosis not only through the *KLF14-SLC7A11* axis but also through metabolic pathways involving glutamine metabolism. Given the emerging evidence that *EZH2* influences various metabolic pathways in cancer [71], this connection between glutamine metabolism, ferroptosis regulation, and the role of *EZH2* could represent an additional mechanism by which *EZH2* promotes tumorigenesis and resistance to ferroptosis. Future studies are warranted to investigate whether *EZH2* might regulate ferroptosis via the *GPX4* pathway in mucosal melanoma, further broadening the therapeutic potential of targeting *EZH2* in ferroptosis-related cancer treatments.

In conclusion, our study underscores *EZH2* copy number gain as a common driver of MM proliferation and ferroptosis resistance, via the *EZH2-KLF14-SLC7A11* axis. This axis offers promising avenues for prognostic assessment and therapeutic intervention in MM.

## 5. Conclusions

This study establishes *EZH2* as a critical driver of MM progression and ferroptosis resistance. We identified the *EZH2-KLF14-SLC7A11* signaling axis as a key regulatory pathway in promoting tumor growth and inhibiting ferroptosis in MM. Our data show that *EZH2* amplification is associated with poor prognosis in MM patients, and that inhibition of *EZH2* sensitizes MM cells to ferroptosis by downregulating *SLC7A11*. These findings offer a potential therapeutic strategy where combining EZH2 inhibitors with ferroptosis inducers could enhance treatment efficacy for MM patients. Targeting this axis may provide a novel approach to improving the clinical outcomes in patients suffering from this aggressive melanoma subtype.

## Figures and Tables

**Table 1:** *EZH2* amplification in melanoma.

Melanoma Subtypes	Number of Cases	Number of Cases with *EZH2* Gain (%)
Acral melanoma	252	58 (23.0)
Mucosal melanoma	148	64 (43.2)
Cutaneous melanoma	147	42 (28.6)
Total	547	164 (30.0)
*p* value		<0.001

**Table 2:** Correlation of *EZH2* gain to clinicopathologic features of mucosal melanoma.

	*EZH2* Genotype		
Clinicopathologic Feature	Gain	No Gain	*p* Value
Age (year)	55.1 ± 10.8	55.3 ± 10.7	0.953
Gender N (%)			0.173
Man	29 (45.3)	28 (33.3)	
Female	35 (54.7)	56 (66.7)	
Ulceration N (%)			0.694
Yes	34 (69.4)	45 (65.2)	
No	15 (30.6)	24 (34.8)	
Primary site			0.615
Head and Neck	25 (39.1)	37 (44.0)	
non-Head and Neck	39 (60.9)	47 (56.0)	
TNM stage N (%)			0.010
I II	16 (25.0)	39 (46.4)	
III IV	48 (75.0)	45 (53.6)	
Mutations N (%)			
NRAS			0.240
Yes	5 (7.8)	2 (2.4)	
No	59 (92.2)	82 (97.6)	
BRAF			1.000
Yes	6 (9.4)	7 (8.3)	
No	58 (90.6)	77 (91.7)	
CKIT			0.473
Yes	2 (3.1)	5 (6.0)	
No	62 (96.9)	79 (94.0)	

**Table 3:** Univariate and multivariate analysis of *EZH2* gain and clinicopathologic factors associated with overall survival in mucosal melanoma.

Variable	OS					
	Univariate			Multivariate		
	HR	95% CI	*p* Value	HR	95% CI	*p* Value
Age (>60 years/≤60 years)	0.83	0.54–1.28	0.40			
Gender (female/male)	0.75	0.50–1.14	0.179			
Primary site (Head and Neck/non-Head and Neck)	1.49	0.83–2.70	0.18			
Ulceration (yes/no)	1.21	0.74–1.99	0.442			
TNM stage (I + II/III + IV)	1.39	0.90–2.14	0.136			
*BRAF* mutation (yes/no)	0.93	0.45–1.92	0.84			
*NRAS* mutation (yes/no)	1.17	0.48–2.89	0.729			
*CKIT* mutation (yes/no)	0.92	0.34–2.52	0.875			
*EZH2* gain (yes/no)	1.65	1.10–2.48	0.017	1.61	1.02–2.56	0.041

## Data Availability

Sequence data that support the findings of this study have been deposited in the Gene Expression Omnibus (GEO) ((https://www.ncbi.nlm.nih.gov/geo/, accessed on 20 June 2024)) with the accession numbers GSE269788 and GSE269789.

## Supplementary Materials

The following supporting information can be downloaded at: https://www.mdpi.com/article/10.3390/cancers16213660/s1, Figure S1: EZH2 expression levels were positively correlated with *EZH2* copy number values. Figure S2: Potential binding sites of *EZH2* in SLC7A11 intron region. Figure S3: Uncropped Western blots. Table S1: Correlation of *EZH2* copy number to *EZH2* protein levels in melanoma. Table S2: Correlation of EZH2 copy number to *EZH2* protein levels in three melanoma subtypes. Table S3: Correlation of *EZH2* copy number gain with clinicopathologic features of melanoma.
