# Supplementary material for: Enhancer of Zeste Homolog 2 Protects Mucosal Melanoma from Ferroptosis via the KLF14-SLC7A11 Signaling Pathway
Source: Cancers (Basel). 2024 Oct 30;16(21):3660. doi: 10.3390/cancers16213660 (PMC11545276; doi:10.3390/cancers16213660)
Supplement: Supplementary file 1 [file cancers-16-03660-s001.zip › Supplementary figures.pdf]

Supplementary Figures

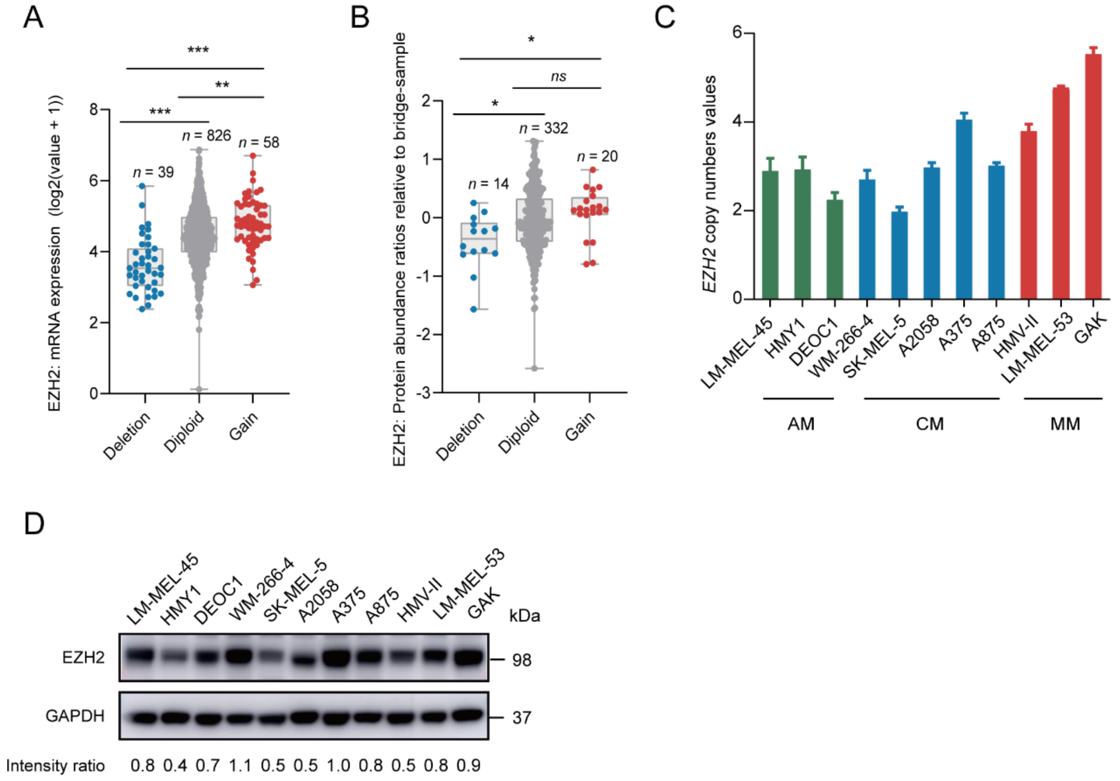

Supplementary Figure S1. EZH2 expression levels were positively correlated with *EZH2* copy number values. (A and B) Correlation of *EZH2* gain status with its mRNA expression (A) and protein (B) expression in different cells from the Cbioportal database. *ns*, not significant, **P* < 0.05, ***P* < 0.01, ****P* < 0.001. (C) *EZH2* copy number value of 10 melanoma cell lines was detected by qPCR. Ribonuclease P (RNase P) as a control gene. The mean ± SEM from 3 experiments was plotted. (D) EZH2 protein levels were determined by western blot assay.

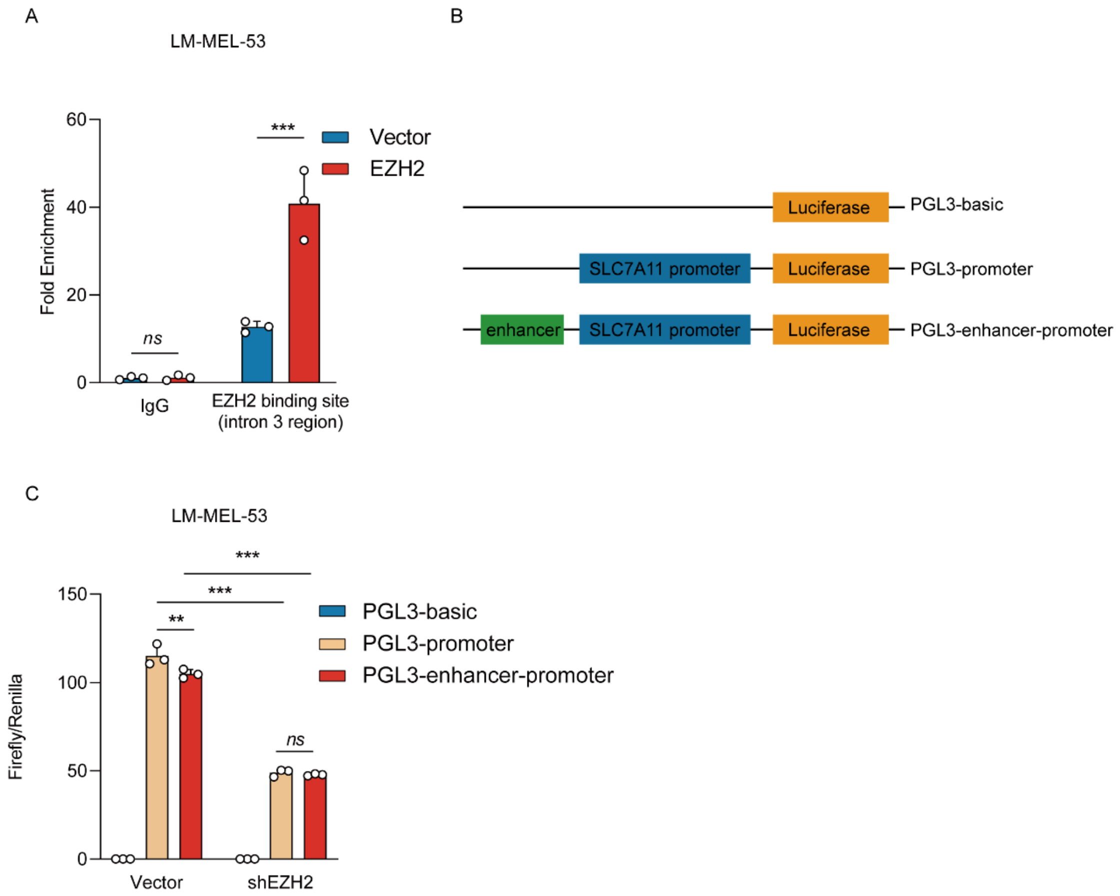

Supplementary Figure S2. Potential binding sites of EZH2 in SLC7A11 intron region. (A) The binding of EZH2 to *SLC7A11* intron region was detected after EZH2 overexpression by ChIP-qPCR. IgG as a negative control. The data are presented as the mean ± SEM. $n = 3$, *ns*, not significant, ***$P < 0.001$. (B) Schematic representation of the luciferase reporter constructs used to assess SLC7A11 promoter activity. (C) Luciferase assay measures *SLC7A11* promoter activity with or without the intron sequence inserted in the absence or presence of EZH2. The data are presented as the mean ± SEM. $n = 3$, *ns*, not significant, **$P < 0.01$, ***$P < 0.001$.

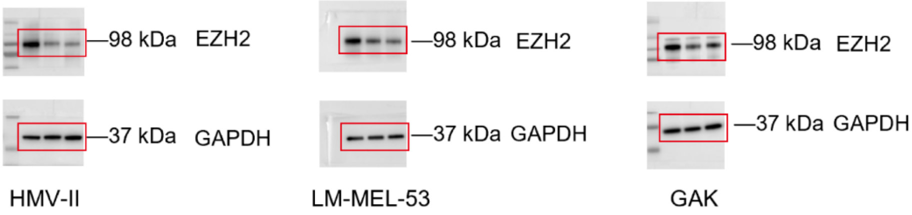

Figure 3A

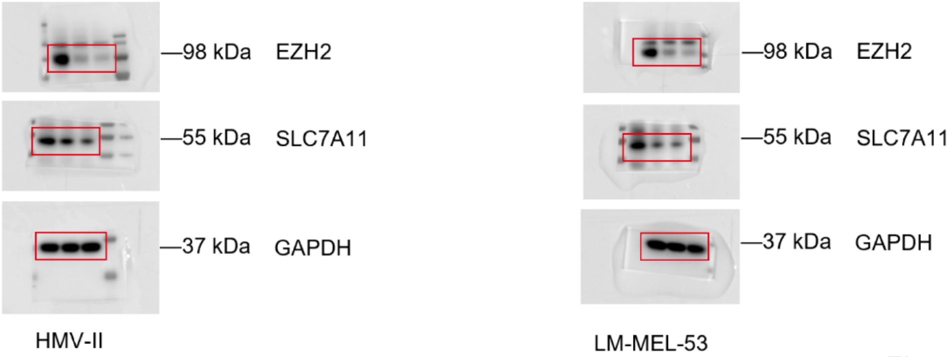

Figure 5D

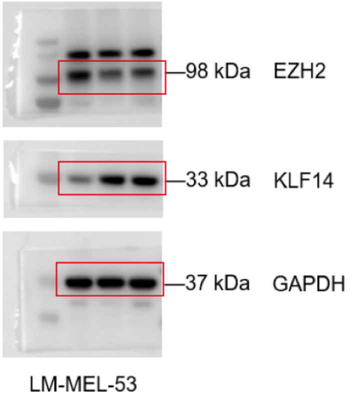

Figure 6D

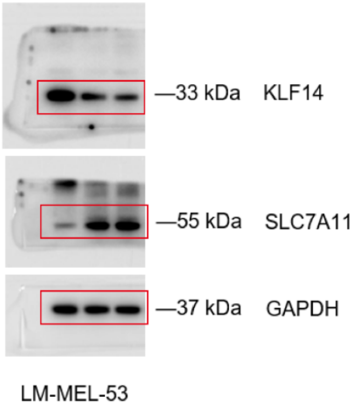

Figure 6E

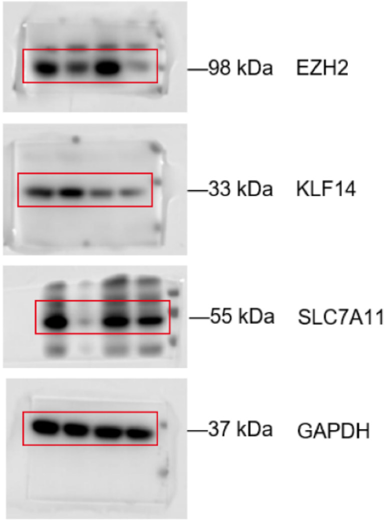

Figure 6I

Figure 6

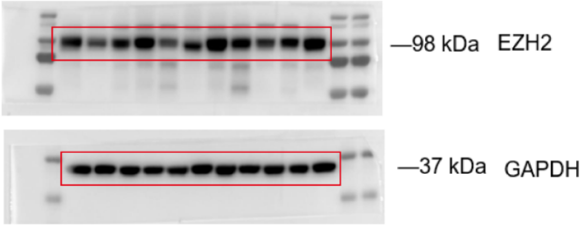

Supplementary Figure S1

Supplemental Figure S3. Uncropped western-blots
