# Supplementary material for: Enhancer of Zeste Homolog 2 Protects Mucosal Melanoma from Ferroptosis via the KLF14-SLC7A11 Signaling Pathway
Source: Cancers (Basel). 2024 Oct 30;16(21):3660. doi: 10.3390/cancers16213660 (PMC11545276; doi:10.3390/cancers16213660)
Supplement: Supplementary file 1 [file cancers-16-03660-s001.zip › Supplementary tables.pdf]

Supplementary Tables

Supplementary Table S1. **Correlation of *EZH2* copy number to EZH2 protein levels in melanoma.**

**IHC scores[a]**	***EZH2* copy number**	
	**Gain**	**No Gain**
**0**	24	92
**1**	10	24
**2**	8	6
**3**	7	13
**Positive rate**	25/48 (52.1)	43/135 (31.9)
***P* value[b]**	0.014	

[a] The signal intensity of immunohistochemistry results were determined by 3 individual pathologists and scored as 0, 1, 2 and 3 with score "0" as negative and score "3" as the strongest.

[b] Significance evaluated by chi-square tests.

Supplementary Table S2. Correlation of *EZH2* copy number to EZH2 protein levels in three melanoma subtypes.

**IHC scores[a]**	***EZH2* gain**		
	**Acral melanoma**	**Mucosal melanoma**	**Cutaneous melanoma**
**0**	56	32	29
**1**	11	14	6
**2**	9	6	4
**3**	4	10	2
**Positive rate**	24/80 (30.0)	30/62 (48.4)	12/41 (29.3)
***P* value[b]**	0.045		

[a] The signal intensity of immunohistochemistry results were determined by 3 individual pathologists and scored as 0, 1, 2 and 3 with score "0" as negative and score "3" as the strongest.

[b] Significance evaluated by chi-square tests.

Supplementary Table S3. Correlation of EZH2 copy number gain with clinicopathologic features of melanoma

	EZH2 copy number		
**Clinicopathologic feature**	**Gain**	**No gain**	***P* value**
Age (year)	51.77 ± 13.44	52.7 ± 12.15	0.425
Gender N (%)			0.190
Man	83 (50.6)	169 (44.1)	
Female	81 (49.3)	214 (55.9)	
Thickness (mm)			0.442
<1	0 (0)	8 (4.3)	
1–2	11 (18.6)	32 (17.1)	
2–4	17 (28.8)	48 (25.7)	
≥4	31 (52.5)	99 (52.9)	
Median (range)	4 (0.25, 30)	4 (1, 18.5)	
Ulceration N (%)			0.116
Yes	77(51.7)	218 (59.2)	
No	72(48.3)	150 (40.8)	
Primary site N (%)			**0.000**
Acral	58 (35.4)	194 (50.6)	
Mucosal	64 (39.0)	84 (21.9)	
CSD	16 (9.7)	45 (11.7)	
Non-CSD	26 (15.9)	60 (15.7)	
TNM stage N (%)			
I	15 (9.1)	34 (8.9)	0.129
II	36 (21.9)	121 (31.6)	
III	48 (29.3)	101 (26.4)	
IV	65 (39.6)	125 (32.6)	
Mutations N (%)			
NRAS			
Yes	9 (5.5)	29 (7.6)	0.465
No	155 (94.5)	354 (92.4)	
BRAF			
Yes	44(26.8)	92(24.0)	0.518
No	120 (73.2)	291 (76.0)	
CKIT			
Yes	5 (3.0)	17 (4.4)	0.635
No	159 (97.0)	366 (95.6)	
